# Analysis of *Pvama1* genes from China-Myanmar border reveals little regional genetic differentiation of *Plasmodium vivax* populations

**DOI:** 10.1186/s13071-016-1899-1

**Published:** 2016-11-29

**Authors:** Xiaotong Zhu, Pan Zhao, Si Wang, Fei Liu, Jun Liu, Jian Wang, Zhaoqing Yang, Guiyun Yan, Qi Fan, Yaming Cao, Liwang Cui

**Affiliations:** 1 Department of Immunology, College of Basic Medical Science, China Medical University, Shenyang, Liaoning 110122 China; 2 Department of Microbiology and Parasitology, College of Basic Medical Sciences, China Medical University, Shenyang, Liaoning 110122 China; 3 Department of Pathogen Biology and Immunology, Kunming Medical University, Kunming, China; 4 Program in Public Health, University of California, Irvine, CA USA; 5 Dalian Institute of Biotechnology, Dalian, Liaoning China; 6 Department of Entomology, The Pennsylvania State University, University Park, PA 16802 USA

**Keywords:** *Plasmodium vivax*, *Pvama1*, Genetic diversity, China-Myanmar border, Malaria

## Abstract

**Background:**

With the premise of diminishing parasite genetic diversity following the reduction of malaria incidence, the analysis of polymorphic antigenic markers may provide important information about the impact of malaria control on local parasite populations. Here we evaluated the genetic diversity of *Plasmodium vivax* apical membrane antigen 1 (*Pvama1*) gene in a parasite population from the China-Myanmar border and compared it with global *P. vivax* populations.

**Methods:**

We performed evolutionary analysis to examine the genetic diversity, natural selection, and population differentiation of 73 *Pvama1* sequences acquired from the China-Myanmar border as well as 615 publically available *Pvama1* sequences from seven global *P. vivax* populations.

**Results:**

A total of 308 *Pvama1* haplotypes were identified among the global *P. vivax* isolates. The overall nucleotide diversity of *Pvama1* gene among the 73 China-Myanmar border parasite isolates was 0.008 with 41 haplotypes being identified (*Hd* = 0.958). Domain I (DI) harbored the majority (26/33) of the polymorphic sites. The McDonald Kreitman test showed a significant positive selection across the ectodomain and the DI of *Pvama1*. The fixation index (*F*_*ST*_) estimation between the China-Myanmar border, Thailand (0.01) and Myanmar (0.10) showed only slight geographical genetic differentiation. Notably, the Sal-I haplotype was not detected in any of the analyzed global isolates, whereas the Belem strain was restricted to the Thai population. The detected mutations are mapped outside the overlapped region of the predicted B-cell epitopes and intrinsically unstructured/disordered regions.

**Conclusions:**

This study revealed high levels of genetic diversity of *Pvama1* in the *P. vivax* parasite population from the China-Myanmar border with DI displaying stronger diversifying selection than other domains. There were low levels of population subdivision among parasite populations from the Greater Mekong Subregion.

**Electronic supplementary material:**

The online version of this article (doi:10.1186/s13071-016-1899-1) contains supplementary material, which is available to authorized users.

## Background

In South and Southeast Asia, Latin America and Oceania, *Plasmodium vivax* is the major malaria species; around 2.5 billion people are living in areas of *P. vivax* transmission [1]. In the Greater Mekong Subregion (GMS) where countries are pursuing regional malaria elimination (www.apmen.org), *P. vivax* is often sympatric with *P. falciparum*, *P. ovale* and *P. malariae* [2], although *P. vivax* has become the predominant species in recent years [3]. With the ability to form hypnozoites that are responsible for relapses, *P. vivax* presents a major challenge for malaria elimination. Within the GMS, malaria is distributed very unevenly; malaria transmission is concentrated along international borders, whereas central plains are mostly free from malaria [3, 4]. The more intensified control efforts in this region have led to a further reduction of malaria incidence, creating isolated areas or pockets of high malaria prevalence separated by areas with extremely low endemicity or malaria-free zones. In China, autochthonous malaria incidence is mostly located in counties bordering with Myanmar, where malaria burden is the highest in the GMS [5, 6]. In these border regions, cross-border human migration as a major source of malaria introduction presents a significant challenge to the malaria elimination course [7, 8]. Since control efforts are expected to have great impacts on the genetic diversity of the parasite populations [9, 10], tracking their spatial and temporal dynamics may provide timely measurement of the progress of regional malaria elimination.

The genetic diversity of antigens in malaria parasites has been extensively studied not only because of their importance as malaria vaccine candidates [11], but also due to their usefulness as molecular markers for differentiating parasite populations. Several *P. vivax* proteins, including Duffy-binding protein (DBP), apical membrane antigen 1 (AMA1), and merozoite surface proteins (MSPs), have been selected as vaccine candidates for their essential functions during erythrocytes invasion and their antigenicity in natural host immune response [12–15]. Among them, AMA1 has been identified as an essential target of the host immune system, and considered an attractive malaria vaccine candidate [15–17]. The *ama1* gene has been extensively studied in a number of *Plasmodium* species [18]. As a type I transmembrane protein, AMA1 is secreted by microneme organelles. Together with RON proteins, AMA1 is involved in merozoite reorientation and tight junction formation during the invasion process [19–24]. Antibodies raised against the AMA1 ectodomain have been shown to inhibit erythrocyte invasion, and AMA1 immunization protects against malaria infection [15, 25–27]. The ectodomain of AMA1 was divided into three subdomains referred to as Domain I (DI), Domain II (DII) and Domain III (DIII) based on the conserved cysteine residues [28]. DI harbors higher levels of genetic variation compared to DII and DIII, suggesting this domain is a target of the host immune system [29]. Within DI of PfAMA1, eight polymorphic amino acids located in the cluster 1 loop (c1L) were identified as the targets of allele-specific, protective immune response [30]. Evidence of diversifying selection was observed in DII of AMA1 in some studies such as in the Sri Lankan parasites, suggesting that this region may also be targeted by host immunity [31, 32]. In addition, serological studies showed that DII is the most immunogenic of the three domains [33]. Due to the highly polymorphic feature of the *ama1* gene, it has been used as a molecular marker for population genetic studies [34, 35].

Although the genetic diversity of *P. vivax ama1* (*Pvama1*) has been extensively studied with samples from Asia, Oceania and South America [36–39], there is a relative lack of data from Southeast Asia, where parasite populations are now being restricted to the international borders as the regional malaria elimination program is unfolding. Thus, in this study, we aimed to investigate the genetic diversity of *Pvama1* gene from the China-Myanmar border area. We wanted to determine whether this potentially isolated parasite population was genetically different from those of other endemic regions. Furthermore, we wanted to learn whether the intensified malaria control efforts resulted in evident reduction of genetic diversity of the parasite population as observed elsewhere in Southeast Asia [9]. Our study revealed a considerably high level of genetic diversity of the *Pvama1* gene in the China-Myanmar border parasite population.

## Methods

### Study sites and isolates

Parasite sample collection was carried out from April 2011 to October 2012 around the township of Laiza, which is located in the northeast Kachin State, along the China-Myanmar border [40]. This region has a subtropical climate with most of the precipitation occurring in June-August (the rainy season). The majority of the local inhabitants are ethnic Kachin (called Jingpo in China) with farming being their major occupation. In recent years, there has been a considerable decline of annual malaria incidence and *P. vivax* has become the predominant parasite species [40, 41]. Like other international borders within the GMS, the China-Myanmar border also has experienced an increase in cross-border migration with large migrant populations engaging in international trade, logging, quarry, plantation and construction activities, which may play an important role for introducing malaria [8, 42]. Filter paper blood spots were collected as our passive malaria case detection efforts in the township hospital and clinics servicing surrounding villages and two camps for internally displaced people. *P. vivax* infections were based on microscopy of Giemsa-stained thick smears. The study protocol was approved by the IRB of Pennsylvania State University and by the Health Department of Kachin.

### DNA extraction, amplification and sequencing of the *PvAMA1* gene

Genomic DNA from dried filter paper blood spots of slide-positive samples was extracted by using a QIAamp DNA Blood Mini kit (QIAGEN, Hilden, Germany). A DNA fragment of the *Pvama1* gene encoding the extracellular region (1,290 bp, nucleotide region (nt) 148–1,437 of the Sal-I sequence; GenBank accession no. AF063138) was amplified and sequenced with six oligonucleotide primers listed in Additional file 1: Table S1. The amplifications were performed in a 20 μl volume reaction containing 1× KOD-Plus-Neo buffer, 200 μM dNTPs, 1 mM MgSO_4_, 250 nM of each primer, 0.4 units of KOD Plus neo polymerase (Toyobo, Osaka, Japan), and 1 μl genomic DNA as template. The reaction was run at 94 °C for 5 min, followed by 45 cycles of 94 °C for 15 s, 56 °C for 15 s, and 68 °C for 90 s, and extension at 68 °C for 5 min. PCR fragments were analyzed by electrophoresis on a 1.2% agarose gels. Direct sequencing of purified PCR fragments was carried out on both strands with primers listed in Additional file 1: Table S1 by using the ABI BigDye™ Terminator Reaction Ready kit (Applied Biosystems, CA, USA).

### Sequence assembling and polymorphism analysis

Of the 78 *P. vivax* infections amplified by PCR, five samples showing dual peaks suggestive of a mixed infection were excluded from further analysis. *Pvama1* was successfully sequenced from 73 samples (sequences submitted to GenBank under accession nos. KX495505–KX495577). A single contiguous 1,290 bp of *Pvama1* (nt 148–1,437 and codons 50–479) was derived for each of the 73 *Pvama1* sequences, which include DI (codons 94–247), DII (codons 265–363) and DIII (codons 388–451). The consensus of the sequences were aligned to the *P. vivax* Sal-I strain by using the CLUSTAL W program in MEGA6.0 [43]. Seven primate-adapted *P. vivax* isolates (Belem, Chesson I, India VII, Indonesia XIX, North Korea, Palo Alto and Simium; GenBank accession nos EU395595–EU395601) were also analyzed as additional reference sequences [44]. Aligned sequences were exported as FASTA alignment for statistical analysis using the DnaSP v5.10.01 software [45]. Sequencing was repeated to confirm singletons. The numbers of segregating sites (*S*), the total number of mutations (*η*), the average number of pairwise nucleotide differences (*k*), nucleotide diversity (π), the number of haplotypes (*H*) and haplotype diversity (*Hd*), and their corresponding standard deviation were computed using the options available in DnaSP v5.10.01 software [45]. The distribution of nucleotide diversity (π) across the DI-III of *Pvama1* gene was analyzed using the sliding window approach.

### Haplotype network construction, linkage disequilibrium (LD) and *F*_*ST*_ analysis

A haplotype network based on the *Pvama1* sequence was constructed by using the NETWORK software Version 4.6.1.3 with the Median-Joining method [46]. A total of 534 publically available *Pvama1* sequences representing seven *P. vivax* populations were retrieved from the GenBank: South Korea [47], India [38], Sri Lanka [32], Thailand [48], Iran [49], Venezuela [50], and Papua New Guinea (PNG) [37]. LD was performed to calculate the minimum number of recombination events (*R*_M_) [51] and to give an estimation of the recombination parameter *C* [52]. The relationship between LD and distance between nucleotide sites was plotted by using indices *D*’ [53] and *R* ^2^ [54]. To assess the proportion of genetic variance due to population subdivision, Wright’s *F*_*ST*_ among populations were calculated by using DnaSP v5.10.01 [45].

### Statistical and phylogenetic analysis

To determine departure from neutrality, Tajima’s *D*, Fu and Li’s D* and F*, and McDonald-Kreitman indices were calculated using DnaSP v5.10.01 and *via* a sliding window method [45]. In Tajima’s *D* test, departure from neutrality is determined by comparing the values of θ (estimated nucleotide diversity) derived from *π* (observed average pairwise nucleotide diversity) and the total number of segregating sites (*S*) [55]. The Fu and Li’s D* and F* tests determine departure from neutrality by differences between estimation of θ derived from the number of singletons and that based on either the total number of mutations (D*) or the average pairwise diversity (F*) [56]. The McDonald-Kreitman test [57] was applied by using a single *P. cynomolgi ama1* sequence (GenBank accession no. X86099; [58]) as the outgroup for comparison with the *P. vivax* populations. It allows for determination of the ratio of synonymous substitutions to nonsynonymous substitutions between and within species. A two-tailed Fisher’s exact test was computed to determine the statistical significance (*P* < 0.05). A phylogenetic analysis of *Pvama1* sequences (nt 322–737 relative to Sal-I strain) with Myanmar samples [59] was performed by the neighbor-joining (NJ) method in the MEGA 6.0 software [43]. The bootstrap method with 1,000 replications was used to assess the reliability of the gene tree.

### Structural modelling of *Pv*AMA1

The potential B cell epitopes of DI-III in PvAMA1 were predicted by using the ABCpred server [60]. In order to consider a given region as a valid linear B-cell epitope, a threshold of 0.7 was used to predict the 16 residues. The prediction of intrinsically unstructured/disordered regions (IURs) in proteins, which is highly desirable in the design of vaccines and diagnostic tests, was carried out using the RONN server [61]. Residues with an output score above 0.5 were regarded as disordered. The overlapped regions between B-cell epitopes and IURs, as well as mutation sites across DI-III were mapped on a three dimensional structure of PvAMA1 (Protein Data Bank ID: 1W8K; [28]). ViewerLite 4.2 was used to visualize the surface distribution of mapped IURs, the polymorphism, and B-cell epitopes on the crystal structure of PvAMA1.

## Results

### Genetic diversity of *Pvama1* among *P. vivax* isolates from the China-Myanmar border

**Fig. 1:**
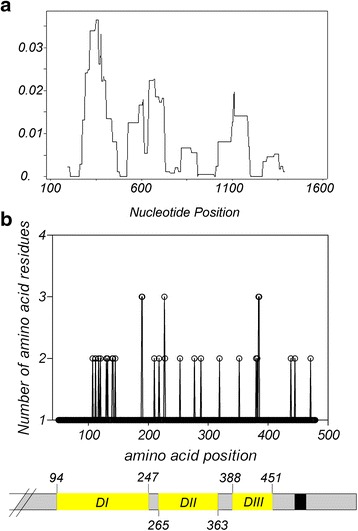
Patterns of nucleotide diversity (**a**) and amino acid polymorphism (**b**) of *Pvama1*. A scheme of the ectodomain of *Pvama1* is shown below. Nucleotide and amino acid positions are after the Sal-I sequence

The 1,290 bp sequence corresponding to the ectodomain (nt 148–1,437 encoding amino acids 50–479 relative to the Sal-I strain) of *Pvama1* was amplified from 73 *P. vivax* samples acquired from the China-Myanmar border. Of the 73 *Pvama1* sequences, there were 41 haplotypes, giving overall haplotype diversity (*Hd*) of 0.958 (Table 1). The average number of pairwise nucleotide differences (*k*) for the entire 1,290 bp sequenced region, DI, DII, and DIII were 10.895, 7.433, 1.385, and 0.419, respectively (Table 1). A total of 46 SNPs was detected, including 13 synonymous and 33 non-synonymous (Table 2). Nucleotide diversity (π) at DI, DII and DIII of *Pvama1* among 73 examined *P. vivax* isolates was 0.016, 0.005, and 0.002, respectively, giving an overall *π* value of the sequenced region of 0.008 (Table 1). A sliding window plot of *π* with a window of 90 bp and step size of 3 bp revealed values ranging from 0.000 to 0.023 (Fig. 1a). The ectodomain contained 43 polymorphic sites, of which four (nt 162, 957, 1377, 1412) were novel compared to previous reports [32, 37, 38, 47–50]. The majority of polymorphic sites were identified in DI (26 sites) and DII (5 sites) as compared to two sites in DIII (Fig. 1b). Of these polymorphic sites, five were trimorphic, while the rest were dimorphic, displaying only two alternative amino acids (Fig. 1b). Within DI, 10 of the 26 *Pvama1* mutations were mapped to the same positions as the *Pfama1* c1, c1L, c2, and c3 clusters. Among them, four mapped to the c1 cluster, and one was located within the *Pvama1* c1L cluster (Additional file 2: Figure S1).

**Table 1:** Nucleotide diversity and summary statistics of *Pvama1* in 73 *P. vivax* isolates from the China-Myanmar border area. The total sequenced region includes codons 50 to 479: Domain I codons 94 to 247 (nt, 280–741), Domain II codons 265 to 363 (nt, 793–1,089), and Domain III codons 388 to 451 (nt, 1,162–1,353)

	*k*	H	*Hd ±* SD	*S*	*η*	*π* ± SD	*θ* ± SD	*D*	D*	F*	*MK*
Total	10.895	41	0.958 ± 0.014	43	46	0.008 ± 0.000	0.007 ± 0.002	0.495	0.772	0.796	0.0001***
D I	7.433	27	0.919 ± 0.022	26	28	0.016 ± 0.000	0.012 ± 0.004	0.912	0.269	0.608	0.004**
D II	1.385	9	0.783 ± 0.031	5	5	0.005 ± 0.000	0.003 ± 0.002	0.780	1.064	1.142	0.064
D III	0.419	3	0.330 ± 0.004	2	2	0.002 ± 0.000	0.002 ± 0.002	0.031	0.712	0.589	0.478

*Abbreviations*: *SD* standard deviation, *k* the average number of nucleotide differences, *H* number of haplotypes, *Hd* haplotype diversity, *S* number of polymorphic (segregating) sites, *η* the total number of mutations, *π* pairwise nucleotide diversity, *θ* the expected nucleotide diversity under neutrality derived from *S. D* Tajima’s *D* test, D* Fu and Li’s D* value, F* Fu and Li’s F* value, *MK* McDonald-Kreitman test

**P < 0.01; ***P < 0.0001

**Table 2:** Genetic diversity of *Pvama1* in eight worldwide populations

Region	Country	*n*	*S*	*η*	*k*	*π* ± SD	*NS*	*SP*	H	*Hd ±* SD	GenBank accession numbers
East Asia	China-Myanmar	73	43	46	10.895	0.008 ± 0.000	33	13	41	0.958 ± 0.014	KX495505–KX495577
	Korea	66	56	56	7.620	0.006 ± 0.000	35	21	27	0.950 ± 0.013	KM230319–KM230384
South Asia	India	10	27	25	10.822	0.008 ± 0.001	20	5	7	0.911 ± 0.077	EF025187–195, 197
	Sri Lanka	23	34	32	10.130	0.008 ± 0.000	27	5	15	0.949 ± 0.028	EF218679–EF218701
Southeast Asia	Thailand	231	52	46	11.927	0.009 ± 0.000	34	12	94	0.934 ± 0.012	FJ784891–FJ785121
West Asia	Iran	29	40	43	12.936	0.010 ± 0.000	34	9	29	1.000 ± 0.009	JX624732–JX624760
South America	Venezuela	73	27	25	9.419	0.007 ± 0.000	19	6	18	0.909 ± 0.016	EU346015–EU346087
Oceania	PNG	102	39	38	10.668	0.008 ± 0.000	31	7	83	0.995 ± 0.002	KC702402–KC702503
	Reference strains	8	43	45	15.607	0.012 ± 0.002	36	9	7	0.964 ± 0.077	EU395595–EU395601
Total		615	130	117	13.497	0.010 ± 0.000	79	38	308	0.988 ± 0.002	

*Abbreviations*: *n* number of samples, *S* number of polymorphic sites, *η* the total number of mutations, *k* the average number of nucleotide differences, *π* nucleotide diversity, *NS* number of non-synonymous polymorphisms, *SP* number of synonymous polymorphisms, *H* number of haplotypes, *Hd* haplotype diversity, *SD* standard deviation

### Genetic diversity of *Pvama1* in the eight worldwide parasite populations

The *Pvama1* sequences obtained from the China-Myanmar border (*n* = 73) were then compared to previously published sequences from seven other geographic regions and primate-adapted *P. vivax* isolates (nt 148–1,437 encoding amino acids 50–479). A total of 117 mutations were identified among the 615 global and reference *Pvama1* sequences, including 38 synonymous SNPs (*SP* SNPs) and 79 non-synonymous SNPs (*NS* SNPs) (Table 2). The majority of the *NS* SNPs (53.2%) were clustered within DI, resulting in a peak nucleotide diversity (*π* = 0.020) for this region (Additional file 3: Figure S2). A total of 308 haplotypes were identified among these global isolates, demonstrating an extremely high level of haplotype diversity (*Hd* = 0.988). Nucleotide diversity was the highest in the Iranian and Thai populations and lowest in the South Korean population (Table 2). Despite that the majority of haplotypes was unique to the China-Myanmar border and Myanmar populations, *Pvama1* sequences from the China-Myanmar border population did not form distinct clades with the Myanmar isolates [59] by phylogenetic analyses (Additional file 4: Figure S3).

### Recombination and linkage disequilibrium

**Fig. 2:**
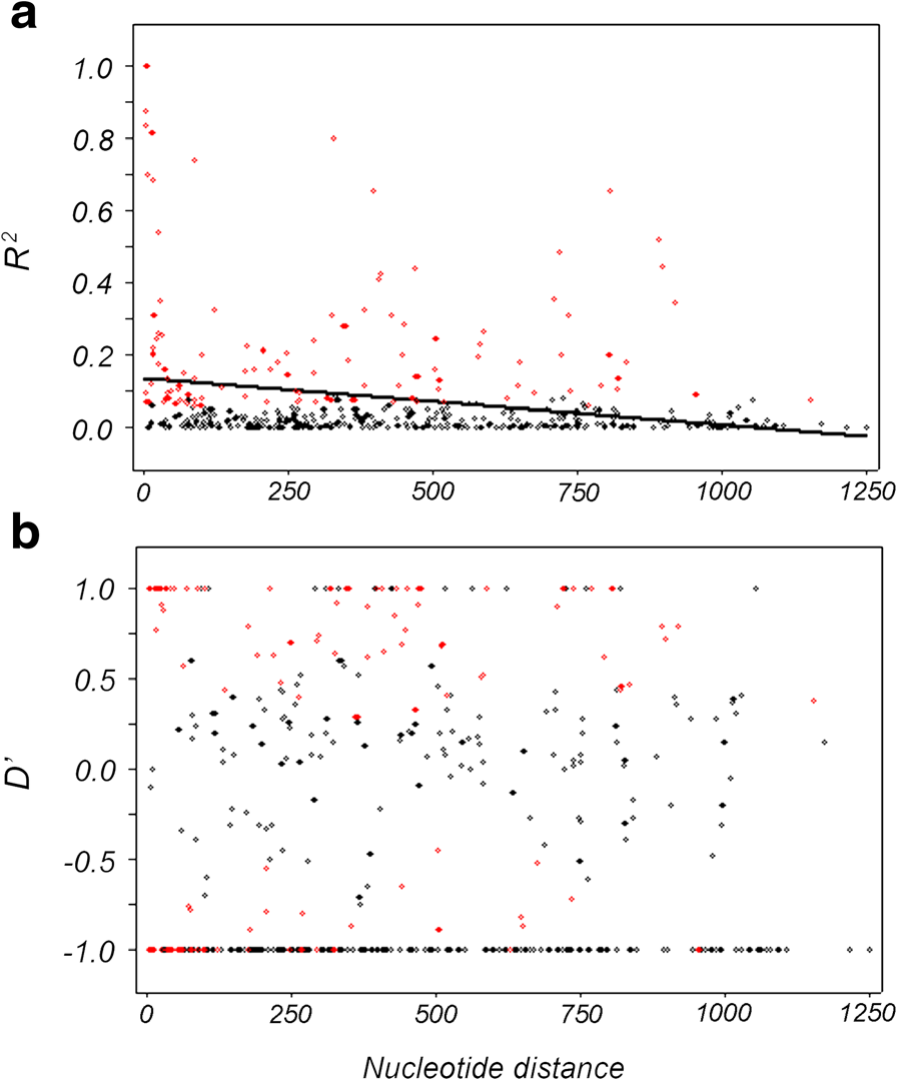
Linkage disequilibrium plots of *R* ^2^ (**a**) and *D’* (**b**) for *Pvama1* gene. Sites with significant linkage (*P* < 0.05) as calculated by Fisher’s exact test are shown as red circles, while all others are shown as black circles. Trace line represents the regression line

Analysis of the ectodomain of *Pvama1* from the China-Myanmar border samples provided estimates of the minimum number of recombination events of six, while values of the recombination parameter *C* between adjacent sites and per gene were 0.043 and 25.5, respectively (Table 3). These values within the China-Myanmar border population were higher compared to estimates from the South Korea, India, Sri Lanka and Venezuela populations, but lower than those of the Thailand, PNG and Iran populations, respectively (Table 3). Figure 2 shows the relationship between *R* ^2^, *D’* with distance between sites. This LD analysis showed a decline of the LD index (*R* ^2^) with increasing nucleotide sites distance within the *Pvama1* gene of the China-Myanmar border isolates, indicating a high meiotic recombination rate (Fig. 2).

**Table 3:** Comparison of different estimates of recombination events in *Pvama1* among worldwide isolates

Locality (no.)	R^a^	R^b^	Rm
China-Myanmar (73)	0.043	25.5	6
South Korea (66)	0.003	1.6	2
India (10)	0.036	21.1	5
Sri Lanka (23)	0.012	7.2	5
Thailand (231)	0.047	27.8	10
Iran (29)	0.067	39.9	8
Venezuela (73)	0.021	12.5	5
PNG (102)	0.054	32	8

*Abbreviations*: *R* ^*a*^ recombinant parameter between adjacent sites, *R* ^*b*^ recombinant parameter for the whole gene, *Rm* minimum number of recombination events, *China-Myanmar* China-Myanmar border population, *PNG* Papua New Guinea population

### *F*_*ST*_ analysis

In order to understand the distribution of diversity across global populations, *F*_*ST*_ values of the China-Myanmar border population and seven worldwide populations with full-length ectodomain sequences were evaluated [45]. A high level of genetic differentiation (*F*_*ST*_ = 0.47) was detected between the South Korean population and the China-Myanmar border population, whereas a low to moderate level of genetic differentiation was detected among other worldwide populations (ranging from 0.03 to 0.24). A moderate range of *F*_*ST*_ values (0.13–0.16) were detected when comparing the China-Myanmar border populations with the India, Iran, Venezuela, and PNG populations, but a much lower genetic difference was identified when comparing the China-Myanmar border population with the Thai population (*F*_*ST*_ = 0.03) (Table 4). Low *F*_*ST*_ values (0.01–0.10) were also observed when comparing part of *Pvama1* DI (nt 322–737) among Myanmar, Thai and China-Myanmar border populations (Additional file 5: Table S2).

**Table 4:** Geographical difference in *Pvama1* sequences across all domains

Locality (*n*)	China-Myanmar	South Korea	India	Sri Lanka	Thailand	Iran	Venezuela
China-Myanmar (73)							
South Korea (66)	0.47						
India (10)	0.13	0.39					
Sri Lanka (114)	0.24	0.54	0.20				
Thailand (231)	0.03	0.47	0.12	0.21			
Iran (29)	0.11	0.37	0.07	0.10	0.12		
Venezuela (73)	0.12	0.48	0.17	0.25	0.17	0.01	
PNG (102)	0.19	0.42	0.06	0.22	0.17	0.26	0.25

*Abbreviations*: *China-Myanmar* China-Myanmar border population, *PNG* Papua New Guinea population

### Evidence of positive diversifying selection on *Pvama1* gene

**Fig. 3:**
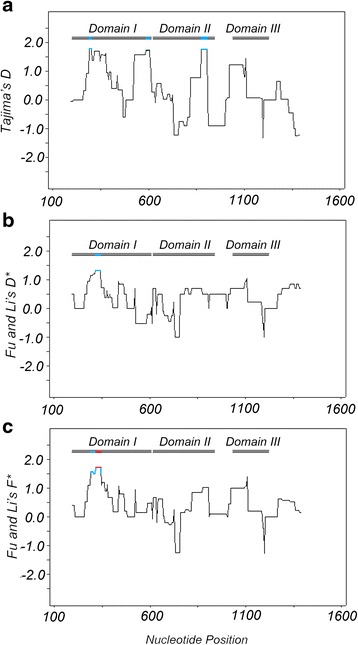
Natural selection on *Pvama1* isolates from the China-Myanmar border area. Sliding-window plots of Tajima’s D test (**a**), Fu and Li’s D* (**b**) and F* (**c**) tests for *Pvama1* were shown. Nucleotide numbers follow those of Sal-I. Window length is 90 bp, and step size is 3 bp. The three domains of AMA1 (Domain I, Domain II, and Domain III) are shown. Blue lines indicate the region with a significant departure from neutrality (*P* < 0.1, one-tailed). Red lines indicate regions with positive values (*P* < 0.05, one-tailed)

To examine whether natural selection contributed to the generation of the diversity in *Pvama1* of the China-Myanmar border *P. vivax* population, Tajima’s *D* and Fu and Li’s D* and F* were performed. The values of Tajima’s *D* and Fu and Li’s D* and F* indices for the entire ectodomain and each domain separately are displayed in Table 1. Even though these values were not statistically significant, the positive value indicates deviation from neutral evolution and the tendency for positive diversifying selection. Meanwhile, a sliding window plot depicted significant positive values in DI, suggesting positive diversifying selection in this region (Fig. 3). Examination of the two sequence sets using a McDonald-Kreitman test showed that the entire sequenced region, particularly within DI, had significantly more non-synonymous substitutions than expected from comparison with *P. cynomolgi* (*P <* 0.0001 and *P* = 0.0037). This result suggests that polymorphisms found for *Pvama1*, especially in the DI, are maintained by diversifying selection, presumably due to host immune pressure.

### Haplotype network reconstruction

**Fig. 4:**
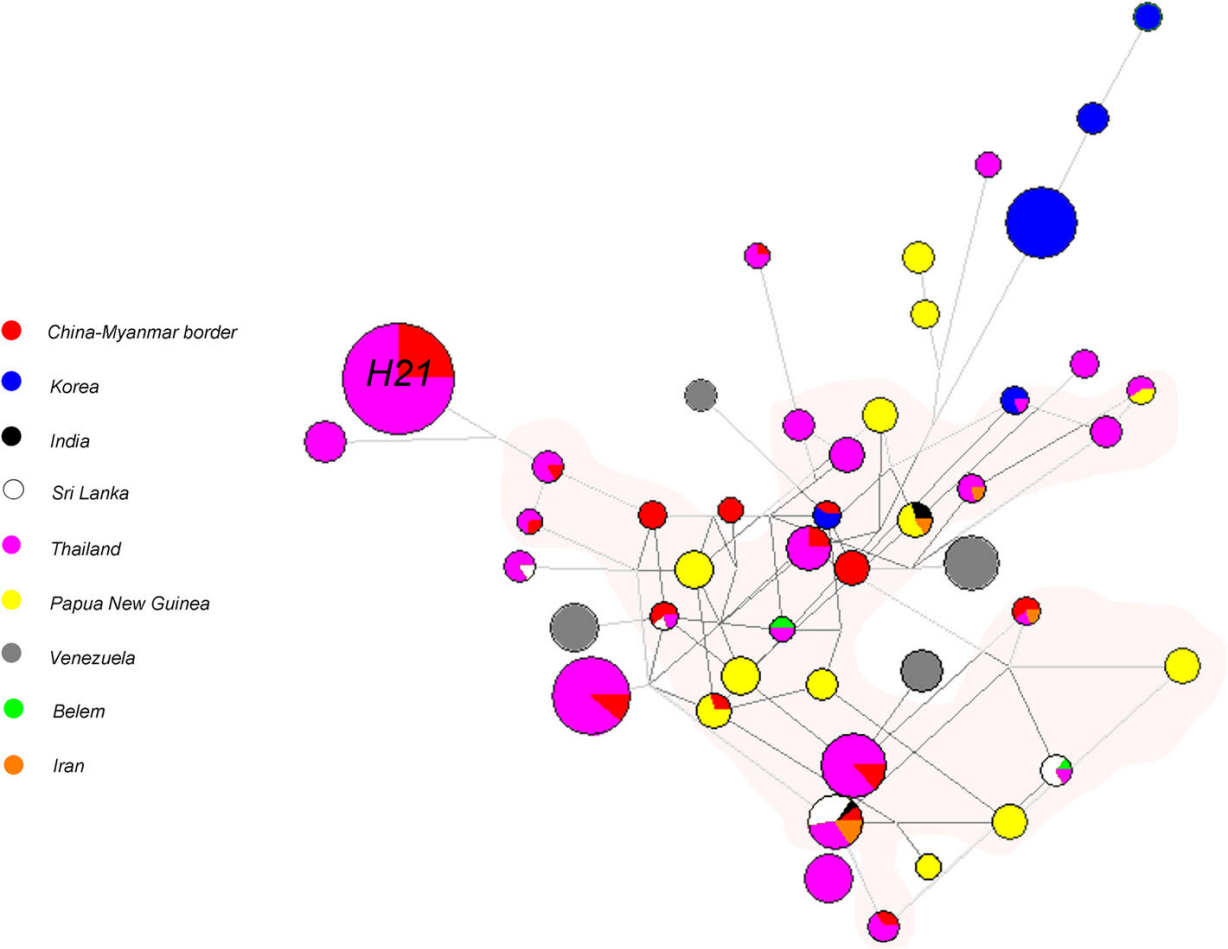
The proportion of *Pvama1* haplotype variations observed in different geographical populations. The size of the pie reflects the frequency of a particular haplotype. The lengths of the lines connecting the pies, measured from their centers, are in proportion to the number of base pair substitutions separating the haplotypes. Color of each pie represents different country. The torso of the haplotype network is shown with light red shadow. *Abbreviation*: H21, haplotype 21

On the basis of the 34 polymorphisms in the *Pvama1* DI global isolates, 162 unique haplotypes were identified in 615 sequences, of which 54.9% was singleton. Individual haplotype prevalence ranged from 0.6 to 45.1%. Of the reference strain *Pvama1* sequences, only the Belem haplotype was detected, but was restricted to the Thai population. No haplotype matched that of the Sal-I strain. Twelve of the 27 China-Myanmar border haplotypes (44.4%) were shared with other populations, of which 91.7% (11/12) were identical to some of the *Pvama1* haplotypes observed in the Thai population. Haplotype 21 is the predominant haplotype both in the China-Myanmar border and Thai populations, with a frequency of 24.7% and 23.8%, respectively. Intriguingly, an independent haplotype analysis performed among the China-Myanmar border, Myanmar, and Thai populations indicates that Haplotype 21 is also the only haplotype shared among these three populations (Additional file 6: Figure S4). Haplotype 10 is shared among the China-Myanmar border, Indian, Sri Lankan, Thai and Iranian populations, with a frequency of 2.7, 10.0, 30.4, 2.6, and 10.3%, respectively. Haplotype 30 is the only haplotype shared between the China-Myanmar border population and the outside Asian population. The haplotype network, drawn by excluding the 89 singletons and ≤ 2% frequency haplotypes from the analysis, showed that clusters from the South American, Oceania, and Asian populations overlapped (Fig. 4).

### Analysis of PvAMA1 structure and antigenicity

**Fig. 5:**
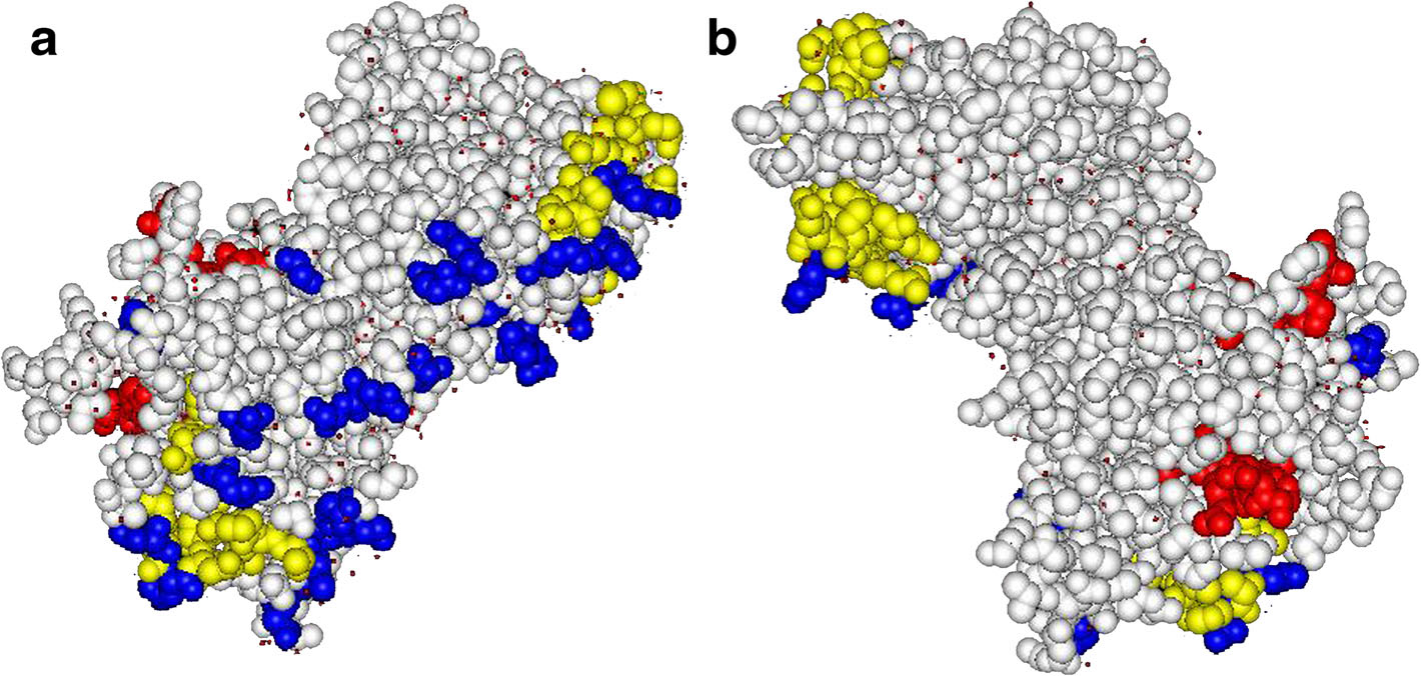
CPK-models of PvAMA1 with IURs, predicted B-cell epitopes, and polymorphic residues across DI-III mapped. Two opposite sides of the same 3-D structure are shown in (**a**) with and in (**b**) without the polymorphic residues. Residues in overlapped regions of B-cell epitopes and IURs with polymorphic residues (aa 121–141, 392–406, and 427–441) are shown in yellow, while overlapped regions without polymorphic residues (aa 171–183 and 291–309) are shown in red. Polymorphic residues across DI-III are shown in pink. Other amino acids are shown in white

To determine the distribution of the B-cell epitopes, IURs, and the polymorphic residues found within the China-Myanmar border population, the modeled structure of PvAMA1 was used. The potential B-cell epitopes were identified across the ectodomain of PvAMA1, which also overlap with the locations of most of the SNPs (Additional file 7: Figure S5). Also, the putative IURs are distributed in five regions (amino acids - aa: 121–141, 171–183, 291–309, 392–406, and 427–441) within all three domains of the PvAMA1 protein with no polymorphic residues detected in aa 171–183 and 291–309. In Fig. 5, the overlap between the putative B-cell epitopes and IURs have been demonstrated in the 3-D model of PvAMA1 in yellow (with polymorphic residues, aa: 121–141, 392–406, and 427–441) and red colors (without polymorphic residues, aa: 171–183 and 291–309), respectively. The polymorphic residues clustered on one side of the PvAMA1 protein. Interestingly, the two overlapping non-polymorphic residues of the B-cell epitopes and IURs are located on the opposing faces of the PvAMA1 protein (Fig. 5).

## Discussion

Consistent with worldwide reports, we detected high genetic diversity in the ectodomain of *Pvama1* sequences obtained in 73 *P. vivax* isolates from the China-Myanmar border. Globally, substantial geographic variability has been observed, with lower diversity in East Asian (Korean, *π* = 0.006 [47]) and South American (Venezuelan, *π* = 0.007 [50]) populations, compared to higher diversity in Southeast Asian (Thai, *π* = 0.009 [48]) and West Asian (Iranian, *π* = 0.010 [49]) populations. As genetic diversity of *P. vivax* populations varies significantly due to transmission intensity [62–64], it is likely that the higher genetic diversity observed in Thailand and Iran was due to higher *P. vivax* transmission intensity than in Korea and Venezuela. The detected high level of genetic diversity in *Pvama1* in parasite population from the China-Myanmar border was not in line with the rapid decline of malaria incidence in recent years. While it is expected that genetic diversity diminishes following the reduction in malaria incidence, the relationship between the two aspects is complex and could vary according to many epidemiological parameters. In Thailand, for example, *P. vivax* population in the western border was highly diverse as compared to being almost clonal in the southern border [9, 10]. It remains to be determined whether these differences were due to different histories of malaria control efforts and levels of parasite introduction from neighboring highly endemic areas. Nevertheless, with the overall increased control efforts in the GMS and more isolated parasite populations on the international borders, future longitudinal tracking of parasite genetic diversity in the local parasite populations as well as in potential sources of parasite introduction will provide answers to these questions. Meanwhile, the use of more than one genetic marker such as a molecular barcode may provide a better resolution of the parasite populations [65].

The LD indices decline rapidly with the increasing distance between sites. Considering the high values of recombination parameter, *C* (*C* = 4*Nr*), these results indicate that recombination is very frequent in the *Pvama1* gene of diverse geographic populations [32, 37, 38, 47–50]. An estimated moderate level of the recombination parameter *C* (both between adjacent site, R^a^ and per gene, R^b^) was detected for *Pvama1* (0.043 and 25.5) in the China-Myanmar border population. This *C* value observed for *Pvama1* is much higher than that of *Pfama1* (0.003 and 3.8) from a sympatric population of *P. falciparum* [66], for which the effective population size (*N*_*e*_) is only 1.3 × 10^3^ when calculated by using the average recombination rate of *P. falciparum* (~6 × 10^-7^ Morgans/base) [67]. We conclude from these data that recombination events in *Pvama1* of the China-Myanmar border population are common, and that this area appears to have a considerably larger effective population of *P. vivax* than *P. falciparum*. In *P. falciparum*, strong LD and low genetic diversity often are observed in low-transmission areas [68]. However, such a spectrum of population structures may not fit for *P. vivax* populations, as we observed LD and high-level haplotype diversity (0.958) of *Pvama1* in the China-Myanmar border, where malaria transmission is low [8]. We speculate that two factors might contribute to the observed high genetic diversity in *Pvama1* in the border regions. The ability to relapse in *P. vivax* increases the prevalence of mixed infections and chances for genetic recombination in the mosquito vectors. In addition, heavy human population flow across the porous international borders further favors mixing of parasite populations and increasing parasite genetic diversity.

Pairwise *F*_*ST*_ comparisons only detected a moderate level of genetic differentiation (*F*_*ST*_ = 0.23) among global populations. This may reflect the functional constraints of this protein and similar balancing selection from human immunity on this protein. Despite this, major geographical genetic differentiation does exist. For instance, the Korean parasite population was drastically different from other parasite populations from the world. The Thai *P. vivax* populations collected from three regions (northwestern, eastern and southern) from 1996 and 2007 also showed significant population subdivision [48], which may be due to temporal and spatial separations among these populations. Later studies confirmed that the southern Thai population had experienced a genetic bottleneck with significantly reduced genetic diversity [9, 10]. Yet, very little genetic differentiation was observed between the combined Thai parasite population and the China-Myanmar border population, despite that our parasite samples were acquired more recently. This demonstrates the persistence of highly diverse *P. vivax* populations with only slight genetic differentiation in different regions of the GMS, suggesting the lack of major gene flow barriers in this region. Together with the fact that similar allelic forms of *Pvama1* are circulating in the Myanmar, China-Myanmar border, and Thai *P. vivax* populations [48, 59], the low genetic differentiation between these regions support the idea that a general malaria elimination or control strategy may be applicable for these endemic regions.

The pattern of polymorphisms observed in the ectodomain as well as DI of the China-Myanmar border *Pvama1* showed that both domains are under diversifying selection, suggesting a significant departure from neutrality as confirmed by the McDonald and Kreitman test. These results are consistent with previous reports of *Pvama1* DI from various global isolates [32, 37, 38, 47–50]. Although DII of *Pvama1* has been reported as highly immunogenic [33, 69], and there is evidence of positive selection in DII of Sri Lankan *P. vivax* populations [32], no evidence was found for diversifying selection on DII and DIII of the China-Myanmar border *Pvama1* as confirmed by neutrality tests, which is in agreement with several other reports [37, 50]. These results suggest that DI is the dominant target of the host immune response.

A total of 308 haplotypes, with a haplotype diversity of 0.988, was identified in global isolates when analyzing the ectodomain of *Pvama1*. This extreme polymorphism indicates that each haplotype might be recognized as immunologically distinct by the immune system. Further observation of *Pvama1* DI showed that only haplotype 30 in the China-Myanmar border is shared with populations outside of Asia, and haplotype 10 is the only one shared among the majority of analyzed Asian populations. Together with the complex network analysis results, covering diversity of *P. vivax* will be a great challenge for a PvAMA1-based vaccine design. Although highly diverse, the observation that the majority of the China-Myanmar border *Pvama*1 haplotypes (44.4%) are shared with Thai populations and that the dominant haplotype (H21) is identical in both populations suggested little differentiation of parasite populations within GMS, and a PvAMA1-based malaria vaccine may be effective in this entire region. Notably, the vaccine strain Sal-I haplotype was not identified in any of the analyzed population in the current study. Thus, the dominant *Pvama1* alleles from field parasite populations should be considered in the future studies.

Compared to the Sal-I sequence, 46 SNPs resulting in 43 amino acid substitutions were identified in the China-Myanmar border *Pvama1*. The majority of the diversity clustered within *Pvama1* DI, as has been reported previously for *P. falciparum* and *P. vivax* [29, 37, 50]. The 90.7% (39/43) polymorphic sites within the ectodomain identified in the China-Myanmar border *Pvama1* are comparable to those previously reported [32, 37, 38, 47–50], indicating that these sites may be under strong natural selection. Four clusters within DI (c1, c1L, c2 and c3) were mapped to the surface of the PfAMA1 protein and are potentially associated with antigenic escape [70, 71]. In *P. falciparum*, the c1 (positions 187 to 231) and c1L (positions 196–207) clusters contain the most polymorphic residues [70, 71]. However, only four *Pvama1* polymorphic sites of the China-Myanmar border isolates were located in the *Pfama1* c1 cluster. These mutation sites are comparable with those observed in the PNG isolates [37], supporting the hypothesis that the presentation of the PvAMA1’s DI loops is different from that of PfAMA1. Structural modeling of PvAMA1 from the China-Myanmar border isolates revealed that all of the polymorphic residues are mapped to one surface of the PvAMA1 protein, suggesting this is the side face exposed to the host immune system [29]. Previous studies by using invasion-inhibitory monoclonal antibodies specific for PfAMA1 revealed that DII of PvAMA1 is a target of the protective immune response. Furthermore, IUR region (aa 290–307) in the DII of PvAMA1 was found to be an important antigenic region during natural human infections [52]. Comparable to these previous reports, amino acid sequence conservation has also been identified in China-Myanmar border isolates, with no polymorphic sites in aa 171–183 (in DI) and 291–309 (in DII) of B-cell epitope and IUR overlapped region have been identified in all 73 sequenced samples. The current results support the inclusion of amino acid sequences aa 291–309 in DII in PvAMA1-based vaccine.

## Conclusions

Our study demonstrated that the genetic diversity of the *Pvama1* in 73 *P. vivax* isolates from the China-Myanmar border area, as well as global isolates, was exceptionally high, and that *Pvama1* DI is the dominant target of positive diversifying selection. Furthermore, a low level of genetic differentiation among the Myanmar, China-Myanmar border and Thai populations suggests high levels of gene flow within the GMS.

## Additional files

Additional file 1: Table S1. Oligonucleotides used for amplification and sequencing of *Pvama1* gene sequences. (DOCX 14 kb)
Additional file 2: Figure S1. Alignment of *P. falciparum*, *P. vivax*, and *P. cynomolgi* AMA1 protein sequences. *P. falciparum* 3D7 (PlasmoDB ID: PF3D7_1133400), *P. vivax* Sal-I (GenBank access no. AF063138) and *P. cynomolgi* (GenBank access no. X86099) were aligned using MUltiple Sequence Comparison by Log-Expectation (MUSCLE, http://www.ebi.ac.uk/Tools/mas/muscle/). Gaps are indicating by dashes. Conserved amino acids among the three analyzed strains are marked by asterisks. Red bold types indicate conserved cysteine residues that divide the ectodomain of AMA1. Domains I-III is shown in grey, green and yellow color, respectively. Boxes indicate c1, c2, and c3 clusters. The c1L cluster region is marked with underlines. Light blue bold type indicates residues that are polymorphic in *P. vivax* of China-Myanmar border isolates. (TIF 2305 kb)
Additional file 3: Figure S2. Patterns of nucleotide diversity and amino acid polymorphisms of *Pvama1*. Sliding window plot of nucleotide diversity (π) and amino acid polymorphism of *Pvama1* ectodomain in 607 global isolates and 8 reference strains were shown. The *π* value was calculated using DnaSP v5.10.01 with window length of 90 bp and step size of 3 bp Domain I is highlighted in red color. Nucleotide and amino acid positions are after the Sal-I sequence. (TIF 183 kb)
Additional file 4: Figure S3. Phylogenetic analysis of China-Myanmar border and Myanmar *Pvama1* sequences. The tree was constructed by analyzing DI (nt, 322–737) with 73 *Pvama1* sequences obtained from China-Myanmar border and 24 *Pvama1* sequences obtained from Myanmar *P. vivax* isolates (GenBank accession nos. FJ157247, FJ157249, FJ157251, FJ157253–FJ157269 and FJ157282–FJ157285) using a neighbor-joining method. The bootstrap method with 1,000 replications was used to construct the gene tree. Red circle indicate sequences obtained from Myanmar. (TIF 1803 kb)
Additional file 5: Table S2. Genetic differentiation (*F*_*ST*_) of the *Pvama1* gene among Thai, Myanmar and China-Myanmar border populations across DI. (DOCX 14 kb)
Additional file 6: Figure S4. The proportion of *Pvama1* haplotypes variation observed in China-Myanmar border, Myanmar, and Thai populations. The size of the pies reflects the frequency of a particular haplotype. The lengths of the lines connecting the pies, measured from their centers, are in proportion to the number of base pair substitutions separating the haplotypes. Color of each pie represents different country. *Abbreviation*: H21, haplotype 21. (TIF 831 kb)
Additional file 7: Figure S5. Location of current study detected SNPs in the predicted B-cell epitopes and IURs in PvAMA1. Predictions of B-cell epitopes and IURs were performed by using ABCpred and RONN server, respectively. The cysteine residues have been shown in red bold. DI = 94–247 aa; DII = 265–363 aa; DIII = 388–451 aa; SNPs in the current study were marked in grey shadow; underlined shows B-cell epitopes; Bold overbars show IURs. (TIF 323 kb)

## Abbreviations

c1L: Cluster 1 loop
DBP: Duffy-binding protein
DI: Domain I
DII: Domain II
DIII: Domain III
*F*_*ST*_: Fixation index
IURs: Intrinsically unstructured/disordered regions
LD: Linkage disequilibrium
*MK*: McDonald Kreitman test
MSP1: Merozoite surface protein 1
NS SNPs: Non-synonymous SNPs
PNG: Papua New Guinea
PvAMA1: *Plasmodium vivax* apical membrane antigen 1
SP SNPs: Synonymous SNPs
