# Supplementary material for: Analysis of Pvama1 genes from China-Myanmar border reveals little regional genetic differentiation of Plasmodium vivax populations
Source: Parasit Vectors. 2016 Nov 29;9:614. doi: 10.1186/s13071-016-1899-1 (PMC5129220; doi:10.1186/s13071-016-1899-1)
Supplement: Additional file 1: Table S1. — Oligonucleotides used for amplification and sequencing of Pvama1 gene sequences. (DOCX 14 kb) [file 13071_2016_1899_MOESM1_ESM.docx]

**Additional file 1: Table S1.** Oligonucleotides used for amplification and sequencing of *Pvama1* gene sequences

|  | Oligonucleotide | 5' to 3' DNA sequence |
| --- | --- | --- |
| PCR | Ppvama1-F | CCTACCGTTGAGAGAAGCACA |
|  | Ppvama1-R | TAGTAGCATCTGCTTGTTCGAT |
| Sequencing | AMA-126F | AAGGGGCCTACCGTTGAGAG |
|  | AMA-534F | TCGTCCTACAGACACCCAGC |
|  | AMA-1466R | TCGTAGCATCTGCTTGTTCG |
|  | AMA-1100R | AATGAGGCAAGTCGGTTTGG |
