# Supplementary figures and images for: Analysis of Pvama1 genes from China-Myanmar border reveals little regional genetic differentiation of Plasmodium vivax populations
Source: Parasit Vectors. 2016 Nov 29;9:614. doi: 10.1186/s13071-016-1899-1 (PMC5129220; doi:10.1186/s13071-016-1899-1)

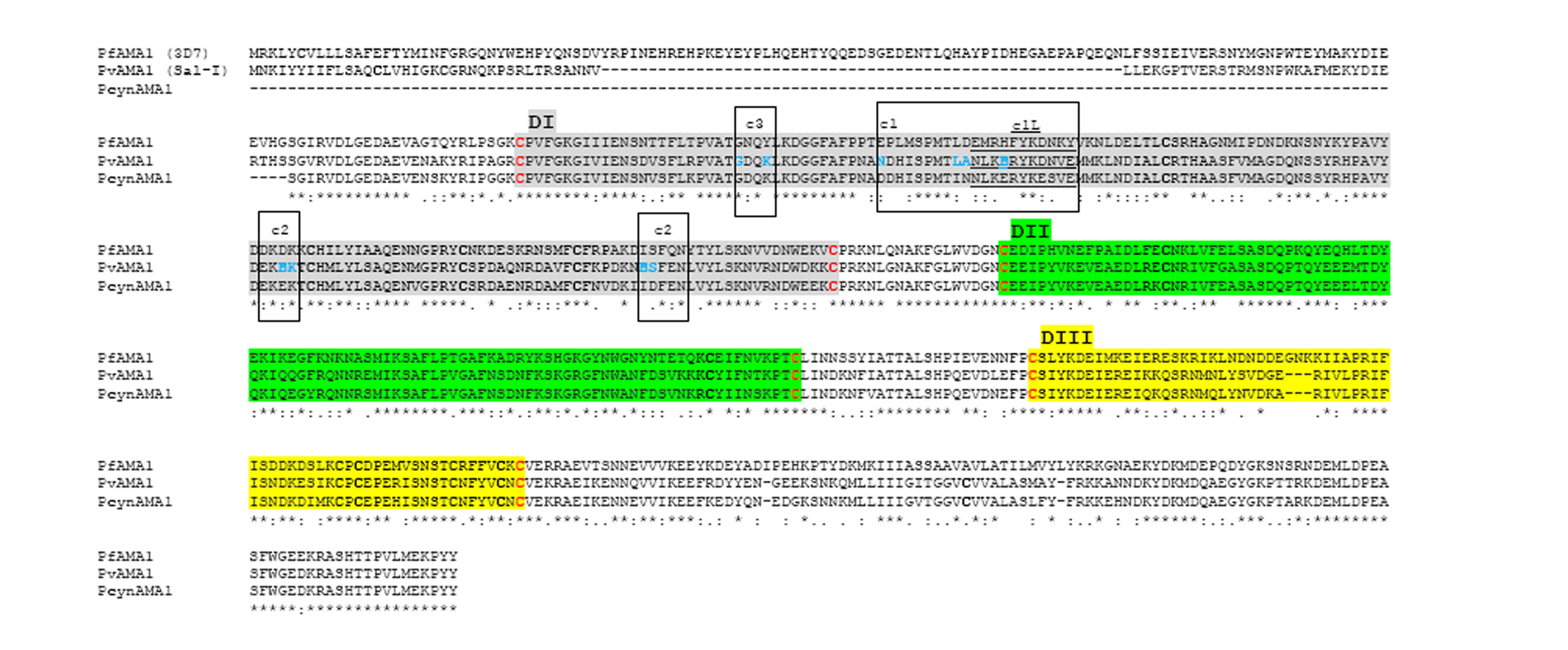

Supplement: Additional file 2: Figure S1. — Alignment of P. falciparum, P. vivax, and P. cynomolgi AMA1 protein sequences. P. falciparum 3D7 (PlasmoDB ID: PF3D7_1133400), P. vivax Sal-I (GenBank access no. AF063138) and P. cynomolgi (GenBank access no. X86099) were aligned using MUltiple Sequence Comparison by Log-Expectation (MUSCLE, http://www.ebi.ac.uk/Tools/mas/muscle/). Gaps are indicating by dashes. Conserved amino acids among the three analyzed strains are marked by asterisks. Red bold types indicate conserved cysteine residues that divide the ectodomain of AMA1. Domains I-III is shown in grey, green and yellow color, respectively. Boxes indicate c1, c2, and c3 clusters. The c1L cluster region is marked with underlines. Light blue bold type indicates residues that are polymorphic in P. vivax of China-Myanmar border isolates. (TIF 2305 kb) [file 13071_2016_1899_MOESM2_ESM.tif]

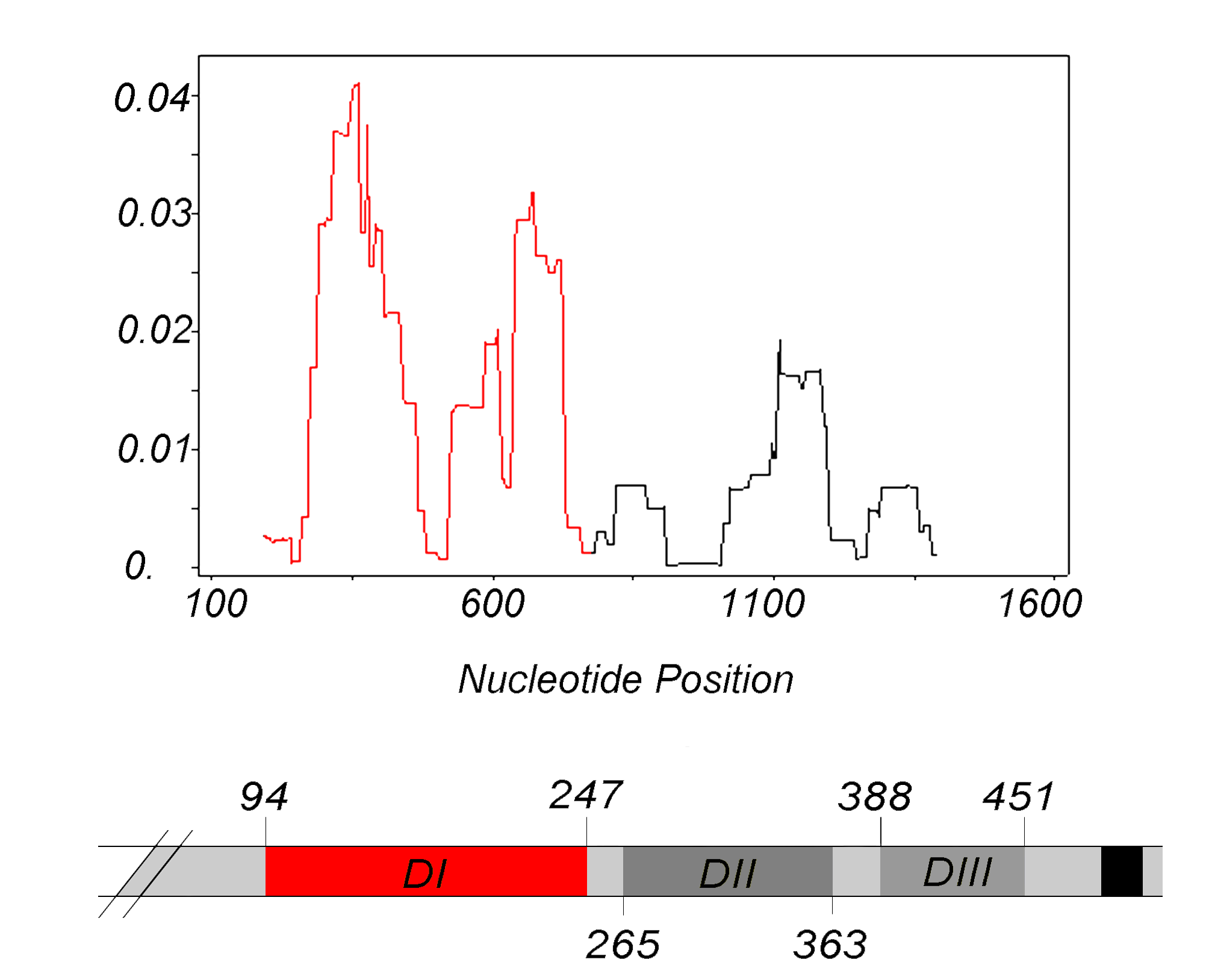

Supplement: Additional file 3: Figure S2. — Patterns of nucleotide diversity and amino acid polymorphisms of Pvama1. Sliding window plot of nucleotide diversity (π) and amino acid polymorphism of Pvama1 ectodomain in 607 global isolates and 8 reference strains were shown. The π value was calculated using DnaSP v5.10.01 with window length of 90 bp and step size of 3 bp Domain I is highlighted in red color. Nucleotide and amino acid positions are after the Sal-I sequence. (TIF 183 kb) [file 13071_2016_1899_MOESM3_ESM.tif]

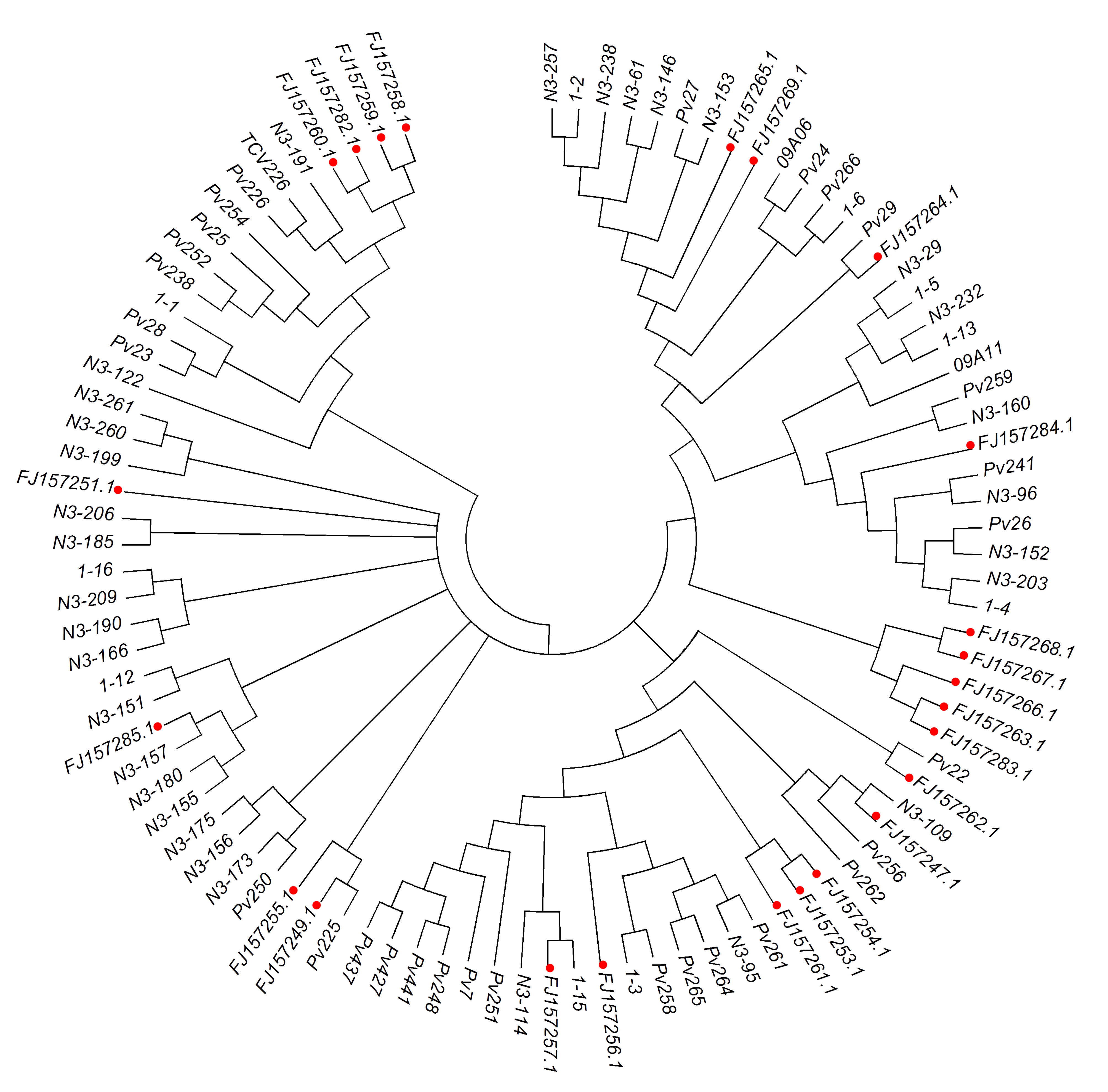

Supplement: Additional file 4: Figure S3. — Phylogenetic analysis of China-Myanmar border and Myanmar Pvama1 sequences. The tree was constructed by analyzing DI (nt, 322–737) with 73 Pvama1 sequences obtained from China-Myanmar border and 24 Pvama1 sequences obtained from Myanmar P. vivax isolates (GenBank accession nos. FJ157247, FJ157249, FJ157251, FJ157253–FJ157269 and FJ157282–FJ157285) using a neighbor-joining method. The bootstrap method with 1,000 replications was used to construct the gene tree. Red circle indicate sequences obtained from Myanmar. (TIF 1803 kb) [file 13071_2016_1899_MOESM4_ESM.tif]

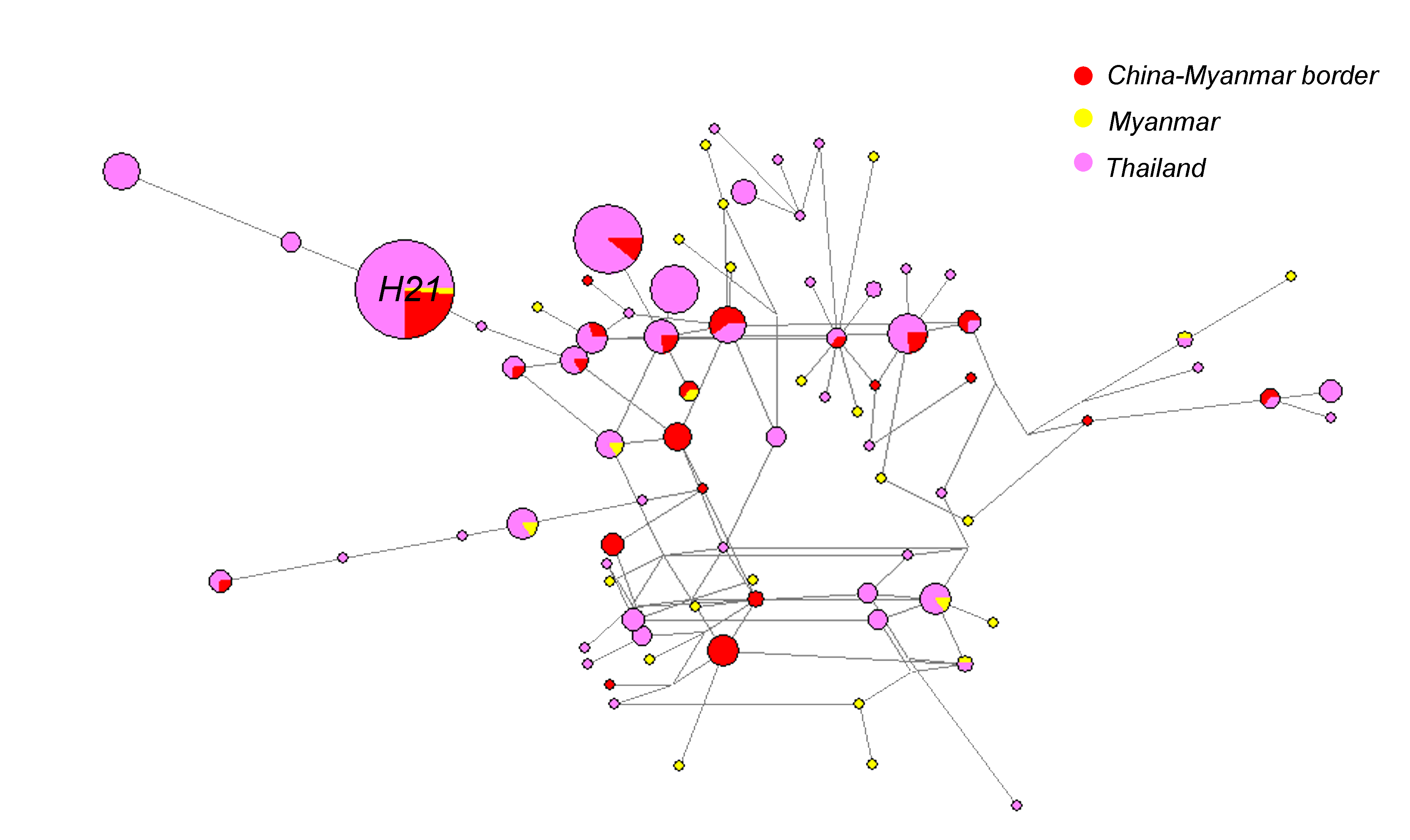

Supplement: Additional file 6: Figure S4. — The proportion of Pvama1 haplotypes variation observed in China-Myanmar border, Myanmar, and Thai populations. The size of the pies reflects the frequency of a particular haplotype. The lengths of the lines connecting the pies, measured from their centers, are in proportion to the number of base pair substitutions separating the haplotypes. Color of each pie represents different country. Abbreviation: H21, haplotype 21. (TIF 831 kb) [file 13071_2016_1899_MOESM6_ESM.tif]

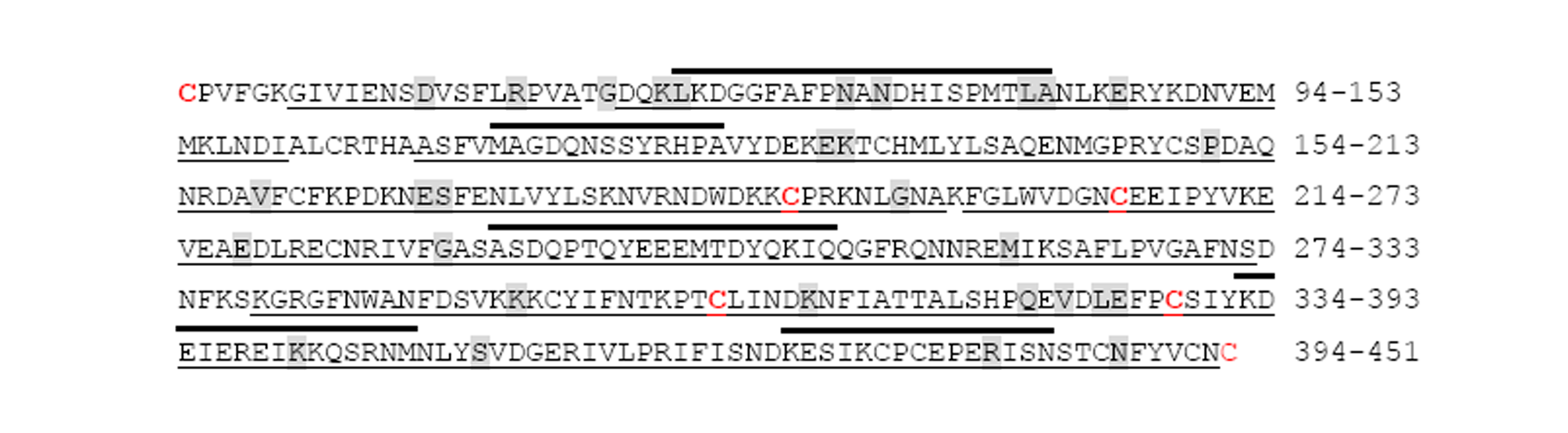

Supplement: Additional file 7: Figure S5. — Location of current study detected SNPs in the predicted B-cell epitopes and IURs in PvAMA1. Predictions of B-cell epitopes and IURs were performed by using ABCpred and RONN server, respectively. The cysteine residues have been shown in red bold. DI = 94–247 aa; DII = 265–363 aa; DIII = 388–451 aa; SNPs in the current study were marked in grey shadow; underlined shows B-cell epitopes; Bold overbars show IURs. (TIF 323 kb) [file 13071_2016_1899_MOESM7_ESM.tif]
