# Supplementary material for: Analysis of Pvama1 genes from China-Myanmar border reveals little regional genetic differentiation of Plasmodium vivax populations
Source: Parasit Vectors. 2016 Nov 29;9:614. doi: 10.1186/s13071-016-1899-1 (PMC5129220; doi:10.1186/s13071-016-1899-1)
Supplement: Additional file 5: Table S2. — Genetic differentiation (F ST) of the Pvama1 gene among Thai, Myanmar and China-Myanmar border populations across DI. (DOCX 14 kb) [file 13071_2016_1899_MOESM5_ESM.docx]

**Additional file 5: Table S2.** Genetic differentiation (*F_ST_*) of the *Pvama1* gene among Thai, Myanmar and China-Myanmar border populations across DI

| Locality (*n*) | China-Myanmar border | Myanmar |
| --- | --- | --- |
| China-Myanmar border (73) |  |  |
| Myanmar (24) | 0.10 |  |
| Thailand (231) | 0.01 | 0.10 |
